# Genetic polymorphisms in immune- and inflammation-associated genes and their association with bovine mastitis resistance/susceptibility

**DOI:** 10.3389/fimmu.2023.1082144

**Published:** 2023-02-23

**Authors:** Muhammad Zahoor Khan, Jingjun Wang, Yulin Ma, Tianyu Chen, Mei Ma, Qudrat Ullah, Ibrar Muhammad Khan, Adnan Khan, Zhijun Cao, Shuai Liu

**Affiliations:** ^1^ State Key Laboratory of Animal Nutrition, Beijing Engineering Technology Research Center of Raw Milk Quality and Safety Control, College of Animal Science and Technology, China Agricultural University, Beijing, China; ^2^ Faculty of Veterinary and Animal Sciences, The University of Agriculture, Dera Ismail Khan, Pakistan; ^3^ Anhui Province Key Laboratory of Embryo Development and Reproduction Regulation, Anhui Province Key Laboratory of Environmental Hormone and Reproduction, School of Biological and Food Engineering, Fuyang Normal University, Fuyang, China; ^4^ Genome Analysis Laboratory of the Ministry of Agriculture, Agricultural Genomics Institute at Shenzhen, Chinese Academy of Agricultural Sciences, Shenzhen, China

**Keywords:** bovine mastitis, immunity and inflammation, genetic markers, polymorphisms, SCS, SCC, inflammatory cytokines

## Abstract

Bovine mastitis, the inflammation of the mammary gland, is a contagious disease characterized by chemical and physical changes in milk and pathological changes in udder tissues. Depressed immunity and higher expression of inflammatory cytokines with an elevated milk somatic cell count can be observed during mastitis in dairy cattle. The use of somatic cell count (SCC) and somatic cell score (SCS) as correlated traits in the indirect selection of animals against mastitis resistance is in progress globally. Traditional breeding for mastitis resistance seems difficult because of the low heritability (0.10-0.16) of SCC/SCS and clinical mastitis. Thus, genetic-marker-selective breeding to improve host genetics has attracted considerable attention worldwide. Moreover, genomic selection has been found to be an effective and fast method of screening for dairy cattle that are genetically resistant and susceptible to mastitis at a very early age. The current review discusses and summarizes the candidate gene approach using polymorphisms in immune- and inflammation-linked genes (*CD4, CD14, CD46, TRAPPC9, JAK2, Tf, Lf, TLRs, CXCL8, CXCR1, CXCR2, C4A, C5, MASP2, MBL1, MBL2, LBP*, NCF1, NCF4, MASP2, A2M, and CLU, etc.) and their related signaling pathways (*Staphylococcus aureus* infection signaling, Toll-like receptor signaling, NF-kappa B signaling pathway, Cytokine-cytokine receptor, and Complement and coagulation cascades, etc.) associated with mastitis resistance and susceptibility phenotypic traits (IL-6, interferon-gamma (IFN-γ), IL17, IL8, SCS, and SCC) in dairy cattle.

## 1 Background

Normally, the mammary gland performs three functions, including the provision of nutrition to offspring in the form of milk, the transfer of immunity from mother to offspring through immunoglobulins in milk, and the provision of protection against microbes (1, 2). Bovine mastitis, the inflammation of mammary glands, occurs when the udder is exposed to any physical injury or pathogenic microorganisms (3). Bacterial pathogens, upon entry, compromise the immunity of the mammary gland and trigger the abnormal regulation of the immune system, followed by inflammatory changes, resulting in mastitis (4).

Based on clinical signs, mastitis can be divided into two types, i.e., clinical and sub-clinical mastitis (5–8). Clinical mastitis is characterized by visible signs of inflammation in the udder and microbiological physical, and chemical changes in the milk (9–11). *Escherichia coli* is one of the major bacteria responsible for acute or clinical mastitis, while subclinical mastitis is caused by the gram-positive *Staphylococcus aureus* (12–14), which is characterized by a marked decrease in milk quantity and quality (15). The other signs of subclinical mastitis are the increase in milk SCC level, which shows the level of leucocytes and epithelial cells in milk (16). Elevated levels of leukocytes are the primary indicator of mammary infection.

An SCC of more than 200,000 cells/mL is an indication of mammary gland infections, while less than 100,000 cells/mL indicates that the cow is uninfected (17). Thus, increased levels of SCC are considered a sign of mastitis. The SCC and/or log-transformed SCC (somatic cell score, SCS) are widely targeted as early indicators of mastitis (3) because of a strong positive genetic correlation (0.6 to 0.90) between mastitis and milk SCC (18–20). The SCC and SCS have comparatively higher heritability than mastitis (21) and are therefore widely targeted in mastitis control by selecting cows with low SCS/SCC (22). However, increased SCC in early lactation can signify the presence of intra-mammary infection, and in many countries, indirect selection against mastitis using milk SCC is practiced (23). However, in the early phases of infectivity, the neutrophil, including the level of inflammatory cytokines, increases more rapidly than the total SCC (24). Moreover, resistance to the pathogenesis of mastitis is a complicated biological mechanism involving various molecules, cells, and pathways (25). That’s why, nowadays, people are more interested in the increasing cells and cytokine levels in milk and blood rather than just the overall SCC, which may show the status of udder health at an earlier stage (26).

The epithelial cells of the inner surface of the mammary gland play a key role in recognizing mastitis-causing pathogens by synthesizing toll-like receptors (TLR2 & TLR4) (27, 28). Upon activation, TLRs further regulate nuclear factor-κB, which translocates into the nucleus and causes the mediation of pro-inflammatory signaling molecules (tumor necrosis factor-α (TNF-α), IL-1β and IL-6, and IL-8) that are essential for the animal’s local and systemic immune reactions (27, 29–31). It has been well-studied that serum cytokines, such as interferon, tumor necrosis factor, IL17, IL6, and IL4, have a key role in inflammatory circumstances, which suggests their possible role in bovine mastitis (32–35). Furthermore, previous studies have also suggested that in addition to SCC and SCS, serum cytokines could also be considered indirect parameters in the control strategies against bovine mastitis (36–38).

The control of mastitis using traditional selection methods is quite challenging due to the low heritability of indirect mastitis resistance phenotypic traits (SCC, SCS, and inflammatory cytokines) and host genetics (39). The use of milk SCC as a surrogate trait for raising mastitis resistance in cattle has achieved limited outcomes (36), thus, the information on molecular markers for mastitis susceptibility/resistance is valuable in identifying genetically mastitis-resistant cattle (40, 41). Due to the limited and slow progress in improving udder health using conventional selection procedures with indirect traits, demand has increased for information on molecular markers to enhance host genetics against mastitis in cattle breeding (4, 42).

Usually, two types of candidate gene approaches (direct and indirect) are used while looking for genetically mastitis-resistant cattle (43–45). The first approach is the use of linked markers (indirect), which are markers that are closed to the gene or QTL that has a significant role in mastitis. The second is the use of functional or direct genetic markers, in which the polymorphisms in genes associated with variation in mastitis resistance phenotypic traits are targeted (43, 46). It is well documented that many genes control mastitis, and all of them contribute considerably to either mastitis resistance or development, so, it is necessary to consider the combination of genes for mastitis resistance because some genes have little effect. It has been documented that the immune and inflammatory response to bacteria is usually regulated by inflammation- and immune-associated genes (47).

So far, several genetic polymorphisms in inflammation- and immune-related genes have been identified for their possible association with mastitis resistance phenotypic traits (33, 34, 37, 40, 41, 46, 48–50). In the current review, we discuss all the polymorphisms in immune- and inflammation-associated genes that are linked to mastitis resistance phenotypic traits (SCC, SCS, IL-6, IL8, IL17, and IFN-gamma). Based on the current review, we suggest that all the highlighted SNPs of the genes discussed could be considered potential genetic markers for mastitis resistance in dairy cattle.

## 2 Methods for collection of literature studies

For this review, we used Google Scholar, Web of Science, and PubMed. Furthermore, we used NCBI to verify the information regarding the genes, such as the number of exons and chromosome location. We only considered data published in the English language and in SCI journals. In addition, we used data that was published between 2000 and 2022. Only the polymorphisms located in exonic regions, promoters, or 5 or 3 untranslated regions of genes were utilized in the current study. Finally, we used the online software, DAVID for biological signaling pathways. The keywords, such as SCS, SCC, inflammatory cytokines, immune- and inflammation-associated genes, and polymorphisms were considered while collecting literature for the current review.

## 3 Genetic polymorphisms of genes associated with mastitis resistance/susceptibility in dairy cattle

The detection of single nucleotide polymorphisms (SNPs) in genes regulating the mammary gland’s innate immunity in response to pathogens has attracted considerable attention within the field of genetic markers in mastitis control research (51–55). Several immune- and inflammation-associated genes and their polymorphisms have been discovered for their association with bovine mastitis susceptibility/resistance (33, 56–60). All the reported polymorphisms in immune- and inflammation-associated genes are summarized in **Table 1**.

**Table 1:** Polymorphisms in genes associated with bovine mastitis resistance phenotypic traits.

Gene	Chromosome Location and No of Exons	Polymorphisms	Location	Nucleotide change	Amino acid change	Biological Function/role in mastitis	Breed (region)	Microbes/ Genetic resistance/ susceptibility to mastitis	Authors
*C5*	Chr8 41 Exons	C5C1(112318429) C5C-2 (112314094) C5C-3 (112308481) C5C-4 (112277923) C5C-5 (112260121) C5C-6 (112250562) C5C-7 (112240847)	Exon-6 Exon-8 Exon-10 Exon-24 Exon-29 Exon-34 Exon-40	G>A C>T G>A G>A G>A G>A A>G	tyr > tyr val>ile thr> thr ser> ser thr>Ile thr> thr gly>gly	Regulates innate immunity in mammary gland against microbial infection, Associated with low milk SCC and SCS	Baladi-Frisian Crossbred (Egypt)	Naturally resistance to mastitis	(25)
*Complement component 4 (C4A)*	Chr23 41 Exons	g.2994 A>G rs132741478	Exon 10	A>G	Methionine and valine at position a362	Showed a link with milk SCS and mastitis resistance	Chinese Holstein cattle (China)	*S. aureus*	(55)
*BRCA1*	Chr19, 23 Exons	c.46126 c.24976 c.25440 c.26198 c.27229 c.27234	Exon-13 Exon-9 Exon-9 Exon-9 Exon-9 Exon-9	G>T T>C A>C C>T A>T A>G	Tyr→Asp Phe→Ser Cys→Arg Synonymous Ile→Lys Glu→Lys	Showed a strong relationship with inflammatory changes and low level of milk SCS	Holstein, Sanhe and Simmental cows (China)	Based on natural resistance/susceptibility to mastitis	(61)
*BRAC1*	Chr19, 23 Exons	c.28300 c.25025	Exon-9 Exon-9	C>A T>A	Thr→Pro Synonymous	Showed a strong relationship with inflammatory changes and low level of milk SCS	Holstein, Sanhe and Simmental cows (China)	Based on natural resistance/susceptibility to mastitis	(62)
*BRAC1*	Chr19, 23 Exons	G43737229T G43761121A	Exon 13 Exon 13	G>T G>A		Functional role in DNA damage repair Coordinates several pathways, with an essential function in cell cycle regulation, protein ubiquitination, transcriptional regulation, and other vital pathways to maintain genome stability Associated with low milk SCC and mastitis	Sahiwal breed (India) Chinese Holstein cattle (China)	Based on natural resistance/susceptibility to mastitis	(55 63, 64)
*MBL1*	Chr26 5 Exons	c.2534 c.2569	Exon-2 Exon-2	G>A T>C	Val→Ile	Indicates a strong link with low milk SCS, Activates an immune response before the induction of antigen-specific immunity	Holstein, Sanhe and Simmental cows (China)	Based on natural resistance/susceptibility to mastitis	(19)
*MBL1*	Chr26 5 Exons	SNP-g.2651 ss172800394	Exon-2	G>A	Val→Ile	Positively correlated with milk SCS in Chinese Holstein, Luxi Yellow, and Bohai Black	Chinese Holstein, Luxi Yellow, and Bohai Black (China)	Based on natural resistance/susceptibility to mastitis	(65)
*MBL1*		g.2651G>A g.−1330G>A	Exon-2 Exon-2	G>A G>A		Associated with milk SCC in Chinese Holsteins	Chinese Holstein cows (China)	Based on natural resistance/susceptibility to mastitis	(66)
*MBL1*		c.2534	Exon-2	G > A		Associated with lower milk SCS in Sahiwal and Hardhenu cattle	Sahiwal breed (India)	Based on natural resistance/susceptibility to mastitis	(67)
*MBL1*		g.2686T>C g.2651G>A	Exon-2 Exon-2	T>C G>A		Associated with lower milk SCS in Sahiwal and Hariana cattle	Hariana and Sahiwal cattle (India)	Based on natural resistance/susceptibility to mastitis	(68)
*MBL2*	Chr26 5 Exons	g.201(ss487448874) g.234(ss487448878) g.235(ss487448881) g.244(ss487448884)	Exon-1	G > A C > A G > A T > C	Arg > Gln Pro > Gln Pro > Gln Asn > Asn	Associated with milk SCS, Activates an immune response before the induction of antigen-specific immunity The low expression may expose the animal to mammary *S.aures* infection Mutation Pro > Gln in MBL with increased NF-κB expression	Chinese Holstein cows (China)	Based on natural resistance/susceptibility to mastitis	(69)
*MBL2*		g.1164 g.1197	Exon-1 Exon-1	G >A C>A	Arg> glu Pro> glu	Linked with lowest milk SCS and mastitis resistance	Chinese Holstein cows (China)	Based on natural resistance/susceptibility to mastitis	(70)
*TLR4*	Chr8, 4 exons	SNP-2021 rs8193069		T>C		Correlated with milk SCS Associated with the regulation of inflammation and immunity by using JAK-STAT signaling upon *S.aureus* infection	Jersey and Jersey x Holstein-Friesian crossbreds (Ireland)	Based on natural resistance/susceptibility to mastitis	(52)
*TLR4*	Chr8, 4 exons	rs8193060	Exon 3	A>G		Positively linked with lowest milk SCC in Brazilian Holsteins	Brazilian Holsteins (Brazil)	Based on natural resistance/susceptibility to mastitis	(71)
*TLR2*	Chr17 6 exons	T385 G	Exon2	T>G		Linked with high milk SCS and mastitis susceptibility	Holstein, Simmental, and Sanhe cattle	Based on natural resistance/susceptibility to mastitis	(72)
*CXCR1*	Chr2 3 Exons	SNP-777 (ss110617059) SNP-1830 SNP-1768 SNP-344, SNP-783	5’ upstream and coding region	C>G A>G T>A T>C C>A		Associated with milk SCS Also plays a key role in innate immunity	Jersey and Jersey x Holstein-Friesian crossbreds (Ireland) Chinese Holstein cattle (China)	Based on natural resistance/susceptibility to mastitis	(52, 73)
*CD4*	Chr5 11 Exons	104010752C/T	Promoter region	C>T		Showed association with lower milk SCS and higher levels of IL6 and IFN-γ and mastitis susceptibility in dairy cattle	Chinese Holstein cattle (China)	Based on natural resistance/susceptibility to mastitis	(33)
*CD14*	Chr7 2 Exons	SNP-1908 (ss5108627)		A>G		Effect on the level of milk SCS and mastitis susceptibility in dairy cattle	Jersey and Jersey x Holstein-Friesian crossbreds (Ireland)	Based on natural resistance/susceptibility to mastitis	(52)
*CD14*	Chr7 11 Exons	g.528 g.612 g.1022	Exon 2 Exon2 Exon2	A/C A/G A/G	(147Ser→Arg) 75Asn→Asp synonymous mutation	Associated with mastitis morbidity	Chinese Holstein cattle (China)	Mastitic cows	(74)
*CD46*	Chr16 17 Exons	(c. 1033 + 2184)	Exon 8	C>T		Plays a central role in the risk of mastitis caused by Streptococcus in dairy cows by using the mechanism of an alternative splicing CD4 controls infection by Streptococcus by activating cell autophagy	Chinese Holstein cattle (China)	*Streptococcus*	(75)
*CARD15*	Chr18 13 Exons	SNP-3168 (rs43710288)	Exon 2	A>T		Significantly regulates innate immunity and shows link with milk SCS	Jersey and Jersey x Holstein-Friesian crossbreds (Ireland)	Based on natural resistance/susceptibility to mastitis	(52)
*IL8*	Chr6, 4 exons	SNP-182 (rs43707839)		A>G		Associated with milk SCS	Jersey and Jersey x Holstein-Friesian crossbreds (Ireland)	Based on natural resistance/susceptibility to mastitis	(52)
*NCF4*	Chr5, 9 exons	SNP g.18475	3′untranslated region(3′UTR)	A>G		Significantly regulates the immune system against pathogens Associated with Mastitis resistance in dairy cows	Chinese Holstein cattle (China)	Based on natural resistance/susceptibility to mastitis	(76)
*NCF4*	Chr5, 9 exons	SNP g.18174	Exon 9			Significantly regulates the immune system against pathogens Associated with higher milk SCS and increased risk of mastitis in cows	Chinese Holstein cattle (China)	Based on natural resistance/susceptibility to mastitis	(77)
*Bovine lactoferrin*	Chr22 and 19 exons	SNP −190	Promoter region	A>G		Show strong link with higher milk SCS	Chinese Holstein cattle (China)	Based on natural resistance/susceptibility to mastitis	(78)
*Transferrin (Tf)*	Chr1 17 Exons	SNP g.13942	Exon 8	T>C	Synonymous	Linked with lower milk SCS in Chinese Holstein, Luxi Yellow, and Bohai Black	Chinese Holstein, Luxi Yellow, and Bohai Black	Based on natural resistance/susceptibility to mastitis	(79)
*CACNA2D1*	Chr4 42 Exons	g.38819398 A526745G	Exon 18 Exon 24	G > A G > A	Asp→ Gly	Correlated with lower milk SCS and mastitis resistance	HF X Sahiwal (India) Sahiwal and Karan Fries cattle (India) Chinese Holstein cattle (China)	Based on natural resistance/susceptibility to mastitis	(80–82)
*BMAP-28, MASP-2*	Chr22 4 Exons	G553A C-86G	Exon 2 Exon 2	G > A	Gly→Ser synonymous	Significantly proportional with lower milk SCS and mastitis resistance	Chinese Holstein cattle (China)	Based on natural resistance/susceptibility to mastitis	(83)
*IL8*	Chr6, 4 exons	SNP -105G>A SNP -A(-180)G	5’ upstream 5’ upstream	G > A G > A		Associated with lower milk SCS and mastitis resistance	Chinese Holstein cattle (China)	Based on natural resistance/susceptibility to mastitis	(84, 85)
*NF-κB* signaling genes *Rel* *p100* *NFKBIZ*	Chr11 Chr26 Chr1	g. 536 g. 94 g.21	Exon 10 Exon 20 Exon 5	C>T G>A C>T	Pro511Ser Arg799Arg Pro152Ser	Associated with the regulation of inflammatory cytokines and suppression of immunity Show link with lower milk SCS and mastitis susceptibility in Chinese Holstein cows	Chinese Holstein cattle (China)	Based on natural resistance/susceptibility to mastitis	(86)
*LBP*	Chr13 15 exons	g.-81 g.11 g.68 g.3034 g.3040 g.3056 g.4619 g.19975	Promoter core region Exon1 Exon1 Exon2 Exon2 Exon2 Exon3 Exon8	C>T T >C G>C G>A A>G T>C G>A G>A	4 Leu→ Ser 23Gly →Ala 36Asp→Asn 38Asn→Asp 43Ile →Thr 67Ala →Thr 282Val→Met	Associated with lower milk SCS and susceptibility to clinical mastitis in Chinese Holstein cows	Chinese Holstein cattle (China)	Based on natural resistance/susceptibility to mastitis	(87)
*LAP3*	Chr16 13 Exons	rs41255599 rs110839532 g.24904	Exon13 Exon13 Exon13	C>T G>T G>C		Enhances immunity and shows a positive relationship with clinical mastitis in dairy cattle	Sahiwal and Karan Fries cattle (India)	Based on natural resistance/susceptibility to mastitis	(88)
*LAP3*		T56C	Exon 12	T>C		Significantly correlated with higher milk SCC and susceptibility to mastitis in dairy cattle	Jersey cows (Poland)	Based on natural resistance/susceptibility to mastitis	(89, 90)
*FAM13A1* *ABCG2* *OPN*	Chr6 29 Exons Chr6 23 Exons Chr6 10 Exons	C28A A86C G391T	Exon 12 Exon 14 Exon 10	C>A A>C G>T		Regulates the higher level of milk SCC and susceptibility to mastitis in dairy cattle	Jersey cows (Poland)	Based on natural resistance/susceptibility to mastitis	(89)
*IL17*	Chr23 3 Exons	rs68268284	Exon 2	T>C		Linked with higher milk SCS and susceptibility to mastitis in dairy cattle	Chinese Holstein cattle (China)	Based on natural resistance/susceptibility to mastitis	(91)
*PGLYRP1*	Chr18 3 Exons	rs68268284	Exon 1	T>C		Linked with lower milk SCS and mastitis resistance	Holstein cows (Poland)	Based on natural resistance/susceptibility to mastitis	(92)
*Clusterin (CLU)*	Chr8 11 Exons	G+15781A C-994T	Exon 17 5′-UTR	G>T C>T		Controls the level of milk SCS and creates resistance against mastitis	Chinese Holstein cattle (China)	Based on natural resistance/susceptibility to mastitis	(93)
*ATP1A1*	Chr3 23 Exons	c-15,739A	Exon 17	C>A		Revealed a significant link with lower milk SCS and mastitis resistance	Chinese Holstein cattle (China)	Based on natural resistance/susceptibility to mastitis	(58)
*PRMT2*	Chr1 12 Exons	C24385T C24375T	3′-UTR 3′-UTR	C/T C/T		Showed higher mRNA expression in infected mammary tissues Associated with mastitis resistance	Chinese Holstein cattle (China)	*S. aureus*	(94)
*TRAPPC9*	Chr14 30 Exons	rs110017379	Exon2	G>T		Linked with lower serum cytokines (IL-6, IFN-g) and higher milk SCC, Showed higher mRNA expression in infected mammary tissues Associated with mastitis susceptibility	Chinese Holstein cattle (China)	Based on natural resistance/susceptibility to mastitis	(33)
*JAK2*	Chr8 26 exons	rs210148032 rs110298451 g.39645396	Exon16 Exon20 Exon20	C/T C/T A>G	Lys > Asx	Correlated with milk SCC IL4, IL-6, IL17, IFN-γ, and mastitis susceptibility	Chinese Holstein cattle (China)	Based on natural resistance/susceptibility to mastitis	(32, 34)
*STAT5B*	Chr11 21 exons	g.43660093	Exon16	T>C		Associated milk SCC, IL-6, and mastitis susceptibility	Chinese Holstein cows (China), Jersey (J) and Achai (A)	Based on natural resistance/susceptibility to mastitis	(34, 95)
*HMGB1*	Chr12 5 exons	g. +2776 A > G	3’-UTR	A > G		Linked with low milk SCC and mastitis resistance	Chinese Holstein cows (China)	Healthy and mastitic cows	(96)
*TLR2*	Chr17 6 exons	T385 G	Exon2	T>G		Linked with low milk SCS and mastitis susceptibility	Holstein, Simmental, and Sanhe cattle	Based on natural resistance/susceptibility to mastitis	(72)

(BMAP-28), Bovine myeloid antimicrobial peptide-28; (MASP-2), mannan-binding lectin-associated serine protease-2; NFKBIZ, NFKB inhibitor zeta; LAP3, Leucineaminopeptidase 3; FAM13A, family with sequence similarity 13 member A; ABCG2, ATP binding cassette subfamily G member 2; OPN, absence of pronuclei; (PGLYRP1), Peptidoglycan Recognition Protein 1 gene; ATP1A1, ATPase Na+/K+ transporting subunit alpha 1; PRMT2, Protein arginine N-methyltransferase 2; (HMGB1), High-mobility group box protein 1.

### 3.1 Innate immune-compartment-associated genes

#### 3.1.1 Bovine peptidoglycan recognition protein 1 (*PGLYRP1*)

Bovine peptidoglycan recognition protein 1 located on chromosome 18, having 3 exons, has a significant role in regulating inflammation, response to infection, and post-infection healing (97). Because *PGLYRP1* works as a receptor for murein peptidoglycans (PGN) of gram-positive and gram-negative bacteria, it is considered a key player in the activation of innate immunity (92). Considering its important role in immunity and inflammation, *PGLYRP1* has been widely targeted in bovine mastitis research (92, 97). A higher expression of PGLYRP1 has been documented in cows with mastitis (36, 97–99). Consequently, the polymorphisms (G + 102C, T -12G) in *PGLYRP1* and SNP-C+4867T in *PGLYRP2* in the exonic region showed an association with milk SCS in dairy cattle (100, 101). Moreover, a study reported that the polymorphism rs68268284 at exon 1 of *PGLYRP1* correlated with milk SCC (92), as mentioned in **Table 1**.

#### 3.1.2 Calcium channel, voltage-dependent, alpha-2/delta subunit 1 (*CACNA2D1*)

Calcium channel, voltage-dependent, alpha-2/delta subunit 1 residing on chromosome 4, containing 42 exons, is another key genetic signature with an important role in clinical mastitis. It has been documented that SNP-G519663A at exon 18 (80), SNP-G519663A, and SNP-A526745G at exon 24 in the bovine *CACNA2D1* gene were significantly linked with lower milk SCS levels and mastitis resistance in dairy cattle (82, 102). Moreover, it has also been noticed that SNP G38819398A at exon 18 (81), variant-T38826986G at exon 19 (103), and SNP-C367284A of *CACNA2D1* (104) were positively linked with milk SCS in Sahiwal and Karan Fries. The above findings show that *CACNA2D1* could be a potential genetic marker against mastitis resistance in dairy cattle.

#### 3.1.3 Mannose-binding lectin (*MBL*)

Mannose-binding lectin is a critical player belonging to the collectin protein family that attaches to a variety of microorganisms and regulates the innate immunity lectin-complement pathway (69). *MBL-A* and MBL-C proteins are encoded by the *MBL1* and *MBL2* genes respectively. The reduced level of *MBL* may expose dairy cattle to various infections, including mastitis (66). Similarly, a study reported a strong correlation of SNP g.2651G>A in the *MBL* gene with milk SCS, suggesting its possible role in mastitis resistance (65). In addition, other studies have also reported the significant association of SNPs in the *MBL* with milk SCS, an essential phenotypic indicator of bovine mastitis (66, 70).

#### 3.1.4 Mannose-binding lectin-associated serine protease 2 (*MASP2*)

Mannose-binding lectin-associated serine protease 2 which is located on chromosome 16 and contains 11 exons, is considered the key protease of the complement system. The *MASP2* gene plays an important role in innate immunity and creates resistance to infections of the mammary gland (105) and is therefore widely studied for its link with mastitis in dairy cattle (83, 105). The polymorphism G553A in *MASP2* was found to be associated with mastitis resistance and lower milk SCC in Chinese Holsteins (83). Similarly, another study also reported that the polymorphisms (g.14047A > C, g.14248T > C, and g.14391C > T) in *MASP2* had a significant correlation with lower milk SCC and mastitis resistance in dairy cattle (105), as shown in **Table 1**.

#### 3.1.5 Lactoferrin (*Lf*)

Lactoferrin is an important, iron-binding glycoprotein member of the serum-transferring protein family, which is produced by the mammary gland and immune cells and distributed in external secretions, such as milk, tears, and polymorphonuclear neutrophil (PMN) cells and plays a crucial role in eliminating bacterial load in certain organs. The Lf has several biological functions, including immunity and bacteriostatic activities, which protect the udder from pathogenic infections (106). A review article comprehensively highlighted the role of *lactoferrin* in bovine mastitis (107). The increased mRNA expression of *Lf* and the elevated level of SCC in the mammary gland with mastitis indicate their role in acute phase response in the mammary gland during mastitis (108). Similarly, another study reported a higher Lf expression during mammary gland infection (109). Recently, several reports have proven the association of genetic polymorphisms in the bovine Lf gene with mastitis susceptibility (53, 110–116). One study documented that SNPs (SNP −190 G>A, −270 T > C, and −190 G >A) in the bovine lactoferrin gene were associated with milk SCS. Furthermore, it has been demonstrated that a higher expression of this gene may render dairy cows susceptible to mastitis (78).

#### 3.1.6 Transferrin (*Tf*)

Transferrin is a β-globulin protein involved in iron ion transportation and plays a significant role in the regulation of innate immunity against microbial pathogens and blocked pathogenic access to iron (117, 118). Being a key player in immunity, *Tf* has been studied for its association with mastitis resistance in dairy cattle (79). In addition, it has been documented that cows with SNP g.13942T>C in Tf show the lowest milk SCS level. Moreover, they noticed a higher expression of *Tf* in the mammary glands of cows with mastitis, which suggests its crucial role in mastitis resistance (79).

#### 3.1.7 Neutrophil cytosolic factor 4 (*NCF4*)

Calcium channel, voltage-dependent, alpha-2/delta subunit 1 is located on bovine chromosome 5 and contains 9 exons. Being a key component of the nicotinamide dinucleotide phosphate (NADPH) oxidase complex, *NCF4* plays an important role in the regulation of biochemical pathways and innate immune responses against microbial infection (119). The role of the NCF4 has been well studied in bovine mastitis research (76, 77). Ju et al. reported that the polymorphism g.18174 A>G on exon 9 of NCF4 showed a noteworthy relationship with high milk SCC levels and is responsible for the mastitis susceptibility of Chinese Holsteins (77). Another study found an SNP on g.18475 A>G in the 3′ UTR of *NCF4*, which was linked with higher levels of milk SCC, suggesting its critical role in mastitis susceptibility (76). It has been well established that, upon challenge with bacteria, the microRNAs (miRNAs) were significantly expressed in the mammary gland, which shows their role in the regulation of host immunity (120). Several studies have documented the role of miRNAs in bovine mastitis and host immune regulation against pathogenic infections (76, 96, 121–125). Consequently, a study reported that the miRNAs-mRNA interaction significantly regulates the expression of NCF4, which is linked with mastitis susceptibility and host immunity (76). Similarly, the SNP g.10766 T>C in *NCF1* caused the aberrant splice variant *NCF1-TV1* production, which showed an association with high milk SCS in dairy cattle (126).

#### 3.1.8 Bovine Alpha-2-macroglobulin (*A2M*) gene

Bovine Alpha-2-macroglobulin gene has 38 exons and is located on chromosome 5. By binding with proteases, *alpha-2-macroglobulin (A2M)* in the plasma and tissues of vertebrates and invertebrates acts as a defense barrier against pathogens (127). Recently, a study briefly explored the role of the *A2M* gene in immunity, inflammation, and infectious diseases (128). As mastitis is characterized by immune depression followed by inflammatory changes, making it a key player in immunity and inflammation, *A2M* has been widely studied in mastitis susceptibility in dairy cattle research (127, 129). The SNP c.3535A> T at exon 29 of the *A2M* gene caused the aberrant splice variant *A2M-AS4* production, which exposes dairy cattle mastitis (127). The Bta-miR-2898 was found to be up-regulated in cows with mastitis when compared with healthy cows. It was experimentally proven that polymorphism at point c.4659_4661delC of the *A2M* gene significantly influences the target bta-miR-2898 binding affinity. This shows that it might be possible that *A2M* has a significant association with mastitis susceptibility (129).

#### 3.1.9 Cluster of differentiation 4 (*CD4*) gene

During mastitis, the inflammatory cells are recruited to the site of infection, where T cells, particularly *CD4* cells, were predominantly observed (130). Polymorphisms in *CD4* and *STAT5B* genes and their link with mastitis resistance phenotypic traits have been well studied (49). The polymorphism in *CD4* at loci g.13598C>T has been identified for its significant association with SCS, which is a crucial indicator of mastitis. Additionally, the study reported that although polymorphisms in *STAT5B* did not show any link with mastitis, when a combination analysis was conducted with *CD4* gene polymorphisms, it was noticed that the combination of both genes showed a considerable effect on SCS (49). In addition, a study has reported a significant association of polymorphisms at point T104010752C and C104028410T in *CD4* and *LAG3* genes, respectively, with milk SCC (131). Based on the published studies, it can be concluded that *CD4* might be a valuable addition to the genetic markers for mastitis resistance in dairy cattle.

### 3.2 Genes encoding pathogen recognition receptors

#### 3.2.1 Toll-like receptors

Toll-like receptors, the family of recognition patterns, are associated with the regulation of innate immunity (132, 133). Recently, various reports have been published on the role of TLRs in mastitis (134–141). The toll-like receptors on mammary epithelial cells show early expression during mammary infection because of interaction with microbial pathogens (142). Upon activation, TLR regulates the expression of several chemokines and pro-inflammatory cytokines, which further facilitate the recruitment of neutrophils and activate innate and acquired immune responses (53). The SNPs within the pattern recognition receptors (PRR) may alter the host response to pathogens and lead to either mastitis resistance or susceptibility (71).

Bovine *TLR4*, which is located on chromosome 8, contains 4 exons, and is a type I transmembrane protein and member of the TLR family, has been identified as a key pattern recognition receptor (PRR) (143). The *TLR4* regulates innate immune system cells when stimulated by pathogen-associated molecular patterns (PAMPs) of foreign microorganisms, including viruses, fungi, and bacteria (144). Furthermore, Wang et al. (143) reported that the LPS, which is considered an essential PAMPS, could be found in most gram-negative and some gram-positive bacteria that interact with *TLR4* in mammary epithelial cells, which is why the higher expression of *TLR4* has been noticed during mastitis in dairy cattle (143). A study reported that SNP- rs8193069 in the *TLR4* gene was significantly associated with higher milk SCC (145, 146). Similarly, another study found that the SNP- 1,397-C-T at exon 3 of TLR4 correlated with milk SCS in Chinese Holstein, Sanhe cattle, and Chinese Simmental (145, 147). Furthermore, Mesquita et al. (71) documented that SNP at point rs8193060 G>A in *TLR4* was significantly associated with the lowest milk SCS level in Brazilian Holsteins. From the above studies, we concluded that *TLR4* has a critical role in mastitis development, and it might, therefore, be considered a potential genetic marker for mastitis resistance.

The polymorphism (SNP -79 T > G) in bovine *TLR1* predisposes dairy cattle to mammary infection due to the excessive activation of NF-κB signaling and poor immune response to pathogens (148). Furthermore, it has been revealed that mutation in *TLR1* also influences the expression of *CXCL8 (IL-8)* (149), *TLR2*, and *IL-6* (34), and high expression of these genes has been reported in the mammary epithelial cells of cows with mastitis. Besides, *TLR1* can establish heterodimers with *TLR2* (150) to widen the recognized ligands. Pant et al. revealed that mutations in *TLR2* and caspase recruitment domain 15 (CARD15) are associated with milk SCS and increase the susceptibility of cattle to mastitis. *TLR2* and *CARD15* are key pattern recognition receptors that play an essential role in the stimulation of inflammatory and immune response (151, 152). A study documented that SNP T385G at exon 2 in the *TLR2* gene is linked with high milk SCS and mastitis susceptibility (72).

#### 3.2.2 Lipopolysaccharide-binding protein (*LBP*)

Lipopolysaccharide-binding protein is another critical gene that has a vital role to play in the innate immune recognition of gram-negative bacteria in dairy cattle. The gram-negative bacteria has been documented to have an essential role in mastitis (87). *LBP* is the critical protein that combines with LPS, a primary plotter from gram-negative bacteria with the ability to activate inflammation in animals. Furthermore, by binding with LPS, the LBP is presented to CD14 ^+^ cells, resulting in the regulation of the TLR4 pathway. The TLR4 pathway further activates the pro-inflammatory response caused by tumor necrosis factor-a (TNF-a), interleukin (IL) -1, or IL-6. In addition, with the help of the soluble form of *CD14*, the LPS-LBP complex also activates the CD14 cell inflammatory response, which shows that *LBP* could significantly contribute to blocking the inflammatory cascade before the release of inflammatory cytokines. Cheng et al. (87) reported that mutations in the *LBP* gene have a significant association with mastitis in dairy cattle, as shown in **Table 1**.

### 3.3 Cytokines encoded genes and their receptors associated with mastitis

The inflammatory cytokines and their associated genes have attracted considerable attention in mastitis research (38). Interleukin-8 receptor α (IL-8RA), coded by the chemokine receptor *CXCR1*, is located on the surface of neutrophil and connects the pro-inflammatory IL-8 with high affinity and is, therefore, targeted widely as a potential marker in mastitis control research (153). Due to the immunosuppressive nature of *S. aureus-*mastitis, IL8RA plays an important role in the control of bovine mastitis by enhancing immunity (154). *IL8*, also called CXC *chemokine ligand 8* (*CXCL8*), is located on chromosome 6 and has 4 exons. *IL8* is considered a potent mediator of inflammation and is also involved in the recruitment of leukocytes to sites of infection (155). The polymorphisms in the *IL8* gene have been the focus of some mastitis research (156, 157). A polymorphism at +472 A>G in *IL8* was reported to be associated with high milk SCC in *S. aureus* mastitis-infected dairy cattle (153). Moreover, the SNP -105G>A in IL8 has been found to be associated with high milk SCS, immunity enhancement, and mastitis resistance (85). It has been documented that the mutation at point +735 G>C could change the amino acid glutamine to histidine in the amino chain of *CXCR2*, which is linked to calcium signaling and G-protein interaction and has a key role in mastitis (158).

*The CXCR1* gene, which coded interleukin 8 receptor α (IL8RA) and has the potential to bind IL8 with high affinity, is located on the surface of neutrophil surface (159, 160). The role of *CXCR1* has been well-studied in mastitis resistance in dairy cattle research (158). Bacteria activate the *CXCR1* after interaction with *TLR4*, which further regulates the NF-κB signaling. The NF-κB translocates to the nucleus, binds with DNA, and causes the expression of the CXCR1 gene. Furthermore, the interaction of *CXCR1* or *CXCR2* with *IL-8* brings changes in neutrophils, which allow their chemotaxis toward the infection in the mammary gland (161), mediates the cell survival and migration, and increases the activity of phagocytosis (162, 163). Several polymorphisms in *CXCR* genes have been identified so far for their possible role in mastitis resistance. Polymorphisms (SNPs -1830AA, -1768TT, and -344TT) (164) and mutation at point (SNP -1768T>A) (165) in the *CXCR1* gene were linked with high milk SCC in dairy cattle. Similarly, another study also reported the significant association of SNP c.337A>G and c.365C>T in *CXCR1* with the milk SCC, suggesting their role in the host response against mastitis (166). A study found that the *CXCR1*+472 variant was significantly linked to milk SCS and increases the susceptibility to *S. aureus-*mastitis in dairy cattle (153). Similarly, other studies have also reported a strong link between *CXCR1* mutations c.980AG, c.735C>G, *CXCR1*+472, *CXCR1+*777, and *CXCR1*−1768 at position 5′ upstream region with milk SCC in dairy cattle (153, 165, 167–169). Based on published research, it can be concluded that *CXCR1* and its reported polymorphisms might be considered potential markers for mastitis resistance/susceptibility in dairy cattle.

The bovine *IL-17* located on chromosome 23, containing 3 exons, is another critical gene studied for its important role in immunity and inflammation pathology (170). Comprehensive reviews have been published on the role of *IL-17* in the mediation of immunity and inflammation (171, 172). Furthermore, it has been demonstrated that the members of the *IL-17* family have a key function in acute and chronic inflammation and have been associated with enhancing the host’s defense against microbial organisms (173, 174). Hu et al. reported that the expression of *IL-17* was significantly up-regulated upon challenging the mammary glands of mice with lipopolysaccharide (LPS). The elevated level of IL-17 is also associated with the regulation of nuclear factor-κB (NF-κB) signaling, which is crucial in mastitis susceptibility. The blockage of IL-17 with the anti-IL-17A antibody has been shown to protect dairy cows from LPS-induced mastitis by suppressing the pro-inflammatory cytokine levels, myeloperoxidase activity, and neutrophil infiltration and NF-κB signaling pathway (175). In 2017, Usman and his co-workers found polymorphism (24392436C/T) in *IL-17F* and mutation (24345410 A > G) in *IL-17A*, which shows an association between the regulation of immunity and inflammation signaling and high milk SCS in both Chinese Holstein and Sanhe cattle (38). Furthermore, the polymorphism (1578A>G) in *IL17A* regulated the milk SCC and, thus, their expression might be a potential marker for mastitis susceptibility (91). The *IL-17A* production was documented during *S. uberis* mastitis (176), and slightly increased expression was also noticed in *S. aureus*-infected cows’ somatic cells (177). Furthermore, an *in-vitro* study illustrated that the *IL-17A* reinforces the ability of mammary epithelial cells (MEC) to resist the consequences of S. aureus (178). Additionally, it has been found that *IL-17F* and *IL-17A* are significantly regulated in mammary tissue in response to *E.coli* (179). Moreover, Roussel and his co-workers experimentally proved that *IL-17 F* and *IL-17A* could play an essential role in regulating host-pathogen relations during mastitis development (179). In addition, a study reported that *IL-17* positively regulates CD4+ T cells to facilitate the immune system against pathogenic infection (180).

### 3.4 The association of genetic polymorphisms in JAK-STAT pathway genes with mastitis

The JAK-STAT has been widely studied for its critical role in immunity and inflammation (181, 182), and evidence indicates that persistent activation of this pathway might lead to many immunity- (183) and inflammation-related diseases (184, 185). Due to its significant role in immunity, cell proliferation, cell differentiation, and inflammation, the JAK-STAT pathway has been widely targeted for therapeutic purposes in several diseases (186). Furthermore, playing a critical role in mammary gland development, any abnormal regulation of the JAK-STAT pathway may disturb normal function, resulting in impairment of mammary gland development and exposure to mammary infections. As mastitis is an immunity and inflammatory-related disease, the JAK-STAT pathway should be explored in mastitis control research (187). *JAK2* and *STAT5A & B* are the key parts of JAK-STAT signaling, which have been recently studied for their association with mastitis resistance (32, 34, 37, 48, 95). Furthermore, it has been noticed that the polymorphism at point 39630048C/T in *JAK2* was associated with interleukin-17 (IL-17) (34), IL-6 and IFN-γ (32). In addition, the SNPs (39652267A/G, 39631175T/C) in the *JAK2* gene were documented for their significant links with milk SCC, IL-6, and IFN-γ (32, 34). The mutation (39631044G/A) in the Jak2 gene was noticed to be significantly associated with milk SCS in Chinese Holstein (34). Moreover, the variation at point 39645396C/T in the *JAK2* gene was linked to milk SCC, IL-6, and IFN-γ (37), while SNP-39631044G/A in *JAK2* was associated with milk SCS (34). Consequently, it is predicted that the SNP (39645396C/T), which regulates the production of inflammatory cytokines, is responsible for changing the amino acid from lysine to asparagines and can, therefore, be targeted as a functional candidate marker for mastitis resistance (37). Other studies also reported a higher expression of *IL-6* in Plasma cell mastitis (PCM), which indicates that the IL-6/STAT3 pathway could play a vital role in the pathogenesis of PCM (188, 189).

A variety of cytokines and growth factors activate *STATs*, which are a family of latent transcription factors. Members of the STATs family are involved in growth, differentiation, survival, and apoptosis. Among the seven members of the *STATs family* (*STAT1*-4, 5a, 5b, and 6) in mammalian cells, *STAT5A* and *STAT5B* are the most closely linked and are the result of duplication (190). *STAT5*, a primary gene of the JAK/STAT inflammation signaling pathway, has an essential role to play in prolactin-induced mammary gland development and is associated with mammary gland development in transgenic mice (191). A few studies noticed a significant association of polymorphism in the *STAT5A* and *STAT5B* genes with mastitis resistance phenotypic traits (34, 37). They documented that polymorphism in *STAT5A* (43046497A/C) was associated with IL-6 and also changed the amino acid isoleucine to valine (34). Similarly, mutation at point 43673888A>G in the *STAT5B* gene was significantly linked to mastitis resistance phenotypic traits (IL-4 and SCC) (37). Bochniarz et al. reported the elevated level of IL-6 and the reduced level of IL-4 in the milk and serum of cows infected with *S. aureus* (192). Khatib et al. noticed that variant 12195T/C in *STAT5A* was significantly linked to a decrease in milk fat and protein percentages as well as levels of SCS in dairy cattle (193). Based on the above-published data, it can be concluded that JAK-STAT signaling plays a key role in immunity and inflammation; thus, the polymorphisms in the genes of JAK-STAT signaling might be valuable additions to the genetic markers for increasing genetic mastitis resistance in dairy cattle.

## 4 Bioinformatic analysis to find the biological signaling pathways for the above-mentioned genes

**Figure 1:**
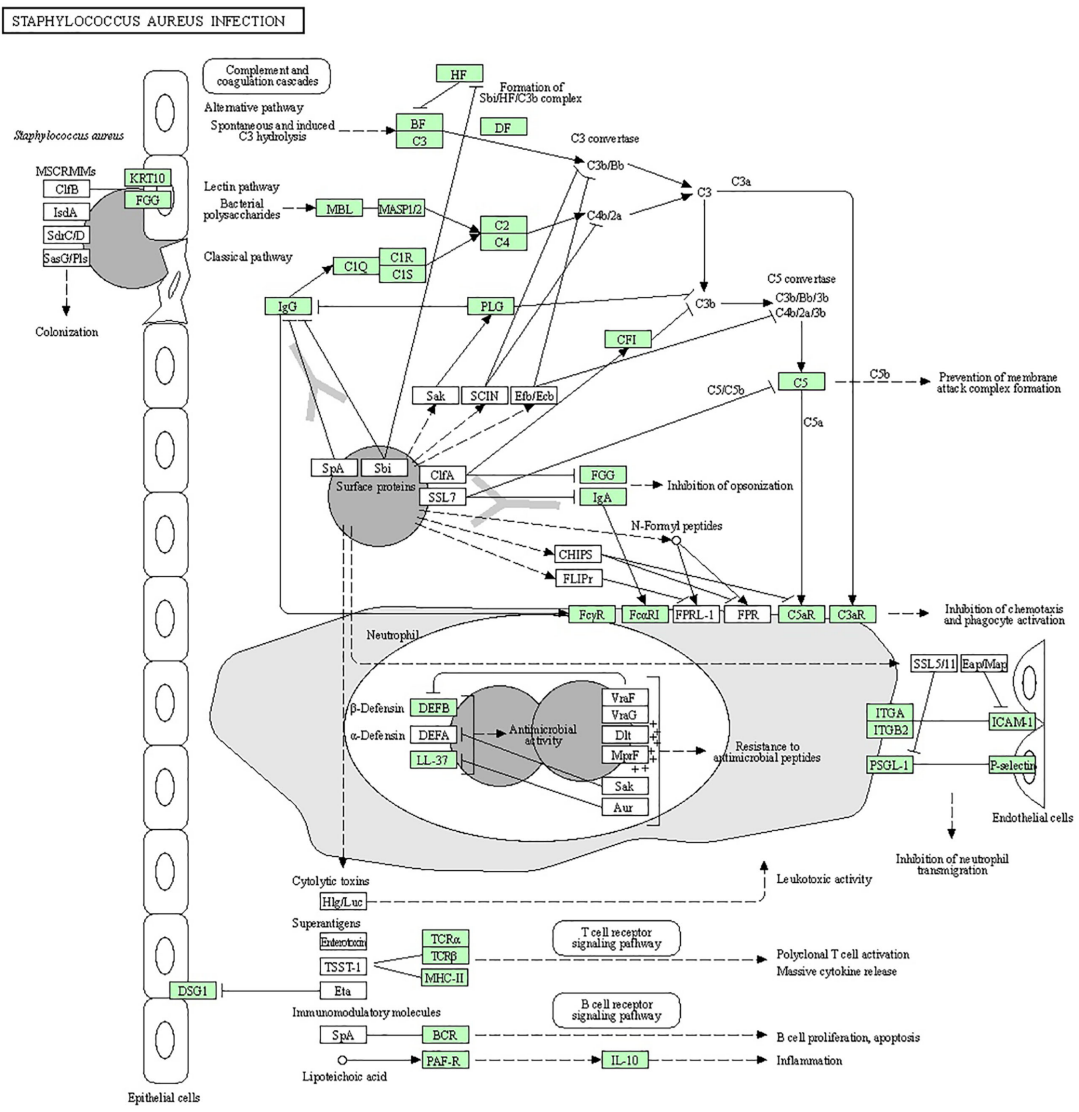
Staphylococcus aureus can cause multiple forms of infections ranging from superficial skin infections to food poisoning and life-threatening infections. The organism has several ways to divert the effectiveness of the immune system: secreting immune-modulating proteins that inhibit complement activation and neutrophil chemotaxis or lysis, modulating sensitivity to cationic antimicrobial peptides (such as defensin) by increasing the positive net charge of its cytoplasmic membrane, and the expression of superantigens that prevent the development of a normal immune response or cause an emetic response when ingested.

**Figure 2:**
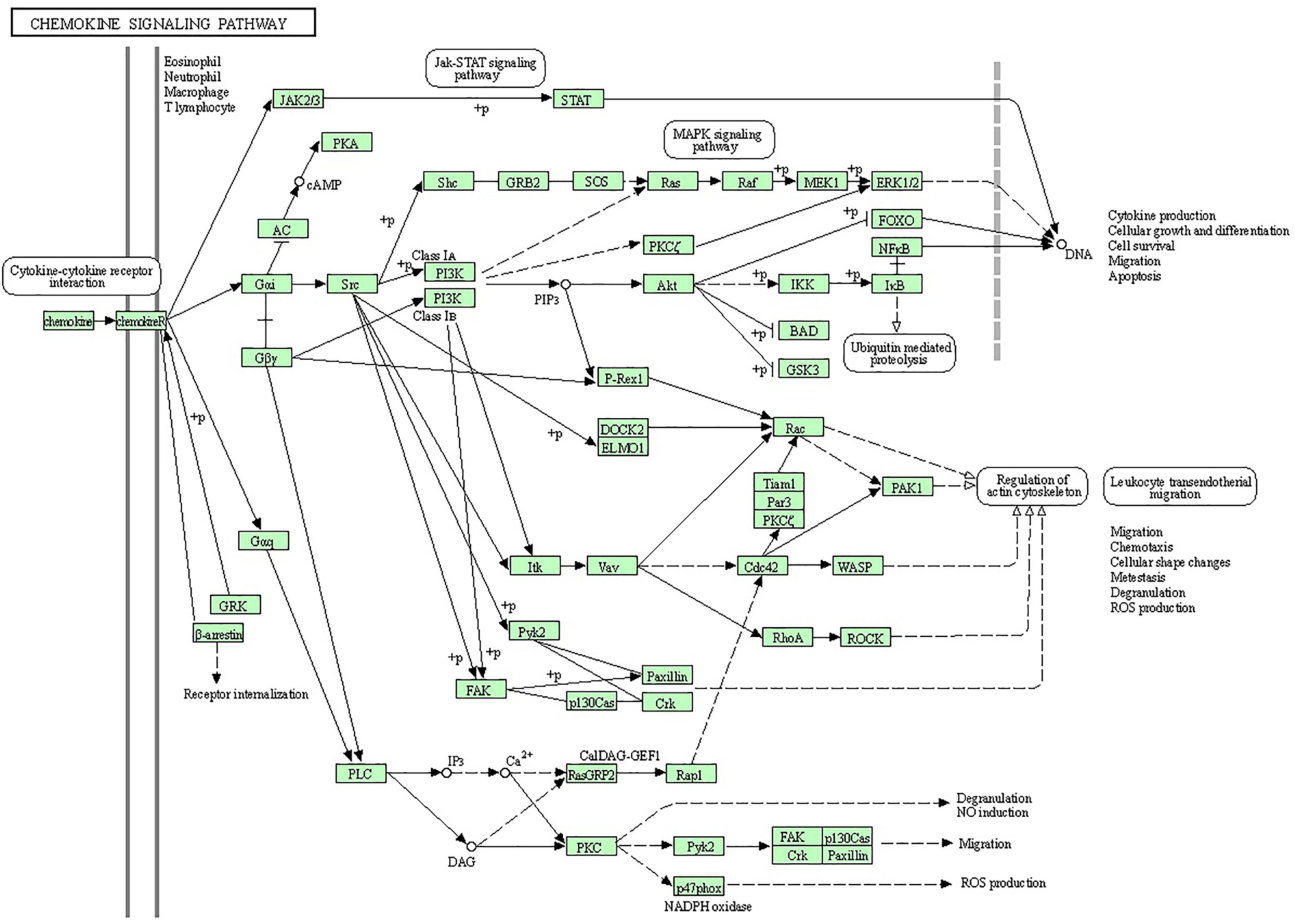
Inflammatory immune response requires the recruitment of leukocytes to the site of inflammation upon foreign insult. Chemokines are small chemoattractant peptides that provide directional cues for cell trafficking and are therefore vital for protective host response. In addition, chemokines regulate a plethora of biological processes of hematopoietic cells, leading to cellular activation, differentiation, and survival. The chemokine signal is transduced by chemokine receptors (G-protein coupled receptors) expressed on the immune cells. After receptor activation, the alpha- and beta-gamma-subunits of G protein dissociate to activate diverse downstream pathways, resulting in cellular polarization and actin reorganization. Various members of small GTPases are involved in this process. The induction of nitric oxide and the production of reactive oxygen species are also regulated by the chemokine signal *via* calcium mobilization and diacylglycerol production.

**Figure 3:**
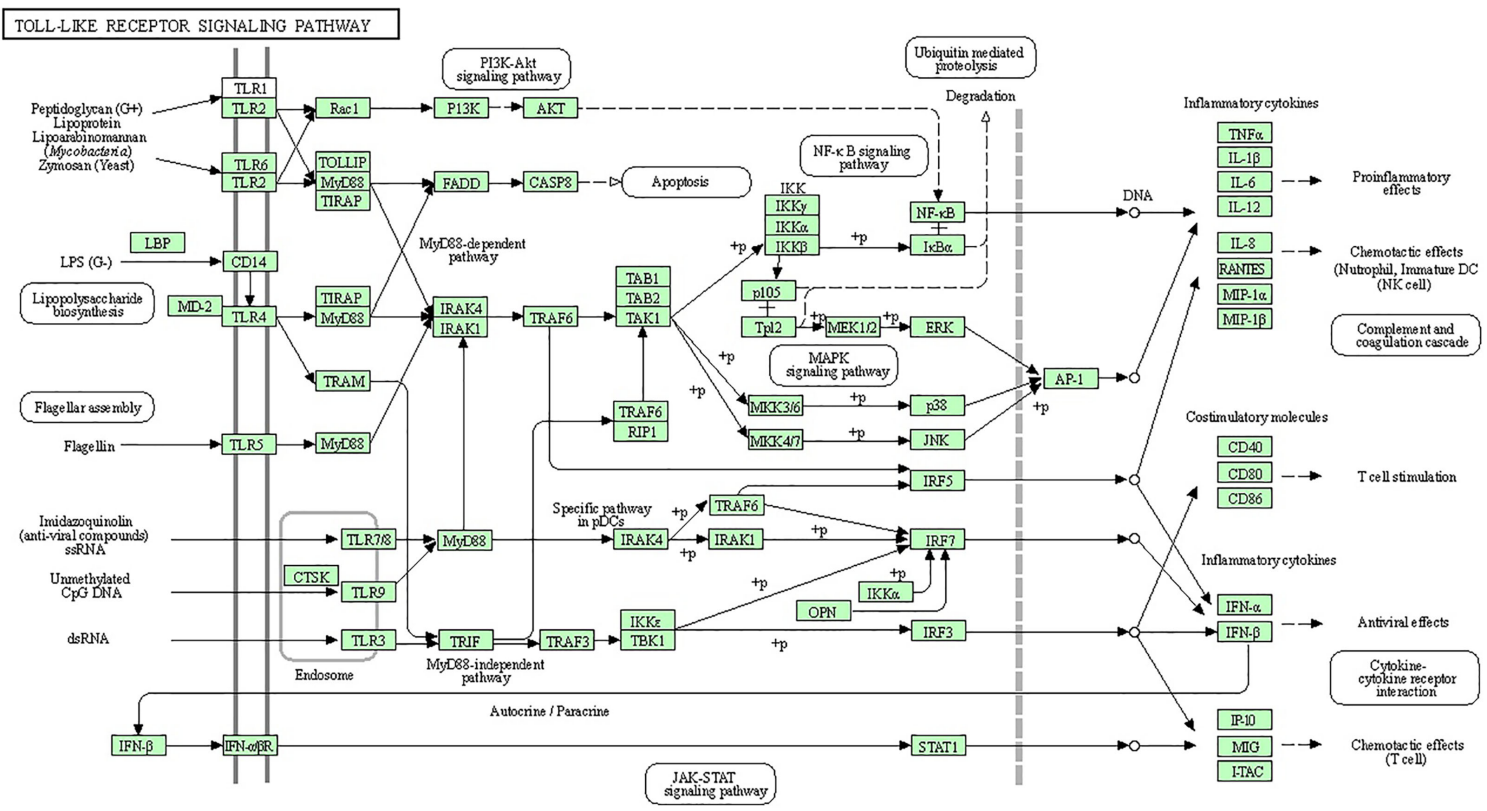
Specific families of pattern recognition receptors are responsible for detecting microbial pathogens and generating innate immune responses. Toll-like receptors (TLRs) are membrane-bound receptors identified as homologs of Toll in Drosophila. Mammalian TLRs are expressed on innate immune cells, such as macrophages and dendritic cells, and respond to the membrane components of Gram-positive or Gram-negative bacteria. Pathogen recognition by TLRs provokes the rapid activation of innate immunity by inducing the production of proinflammatory cytokines and the upregulation of costimulatory molecules. TLR signaling pathways are separated into two groups: a MyD88-dependent pathway that leads to the production of proinflammatory cytokines with the quick activation of NF-KB and MAPK and a MyD88-independent pathway associated with the induction of IFN-beta and IFN-inducible genes and the maturation of dendritic cells with slow activation of NF-KB and MAPK.

Bioinformatics analysis was performed to discover the significantly regulated biological signaling pathways of the above-mentioned genes. For this purpose, we collected all the genes (mentioned in **Supplementary Table 1**) with their Ensembl IDs (already discussed in the current review article) and uploaded them to the online DAVID software (https://david.ncifcrf.gov/tools.jsp) (194). Through bioinformatics analysis, we reported several immunity and inflammatory signaling pathways (**Supplementary Table 2**), of which, staphylococcus aureus infection, chemokine signaling pathway, toll-like receptor signaling pathway, complement and coagulation cascades, cytokine-cytokine receptor interaction, and NF-kappa B signaling pathway were found for their involvement in mastitis (**Table 2**). In addition, the regulation mechanisms of staphylococcus aureus infection, chemokine signaling pathway, and toll-like receptors signaling pathways are shown in **Figures 1**–**3** respectively.

**Table 2:** Immune- and inflammation-associated genes and their biological signaling pathways linked with bovine mastitis.

Biological signaling pathways	Genes involved in biological signaling pathways	References
bta04610:Complement and coagulation cascades	C4A, C5, MASP2, A2M, CD46, CLU, MBL1, MBL2	(187, 195); current analysis
bta04145:Phagosome	NCF1, NCF4, CD14, TLR4, MBL1, MBL2, TLR2	(196), current analysis
bta05150:Staphylococcus aureus infection	C4A, C5, MASP2, MBL1, MBL2	(187, 196); current analysis
bta04620:Toll-like receptor signaling pathway	CXCL8, LBP, CD14, TLR4, TLR2	(187, 195); current analysis
bta04613:Neutrophil extracellular trap formation	C5, NCF1, NCF4, HMGB1, TLR4, TLR2	current analysis; (197, 198); current analysis
bta04658:Th1 and Th2 cell differentiation	STAT5A, STAT5B, CD4, JAK2	199; current analysis
bta04064:NF-kappa B signaling pathway	CXCL8, LBP, CD14, TLR4	(40, 187, 195); current analysis
bta04659:Th17 cell differentiation	STAT5A, STAT5B, CD4, JAK2	(199); current analysis
bta04060:Cytokine-cytokine receptor interaction	CD4, CXCL8, CXCR1, CXCR2	(187, 195); current analysis

## 5 Conclusion

Altogether, we concluded that an appropriate approach to identifying genetically resistant animals would be to study the association of host genetics with mastitis susceptibility and resistance, thus, genetic marker selection was determined as the best approach to screening genetically mastitis-resistant cattle. In the current review, we have highlighted potential genetic polymorphisms in inflammation- and immune-associated gene markers that are significantly associated with mastitis resistance/susceptibility in dairy cattle. The highlighted polymorphisms in immune- and inflammation-associated genes could be considered potential biomarkers in bovine mastitis control research.

## Author contributions

MZK, JW, SL and ZC designed the study and wrote the manuscript; ZC and SL supervised the manuscript; MM, QU, AK, YM, JW, TC, IMK, MZK and SL helped in the collection of data resources and editing of final version of the manuscript. All authors contributed to the article and approved the submitted version.
