# Supplementary material for: Genetic polymorphisms in immune- and inflammation-associated genes and their association with bovine mastitis resistance/susceptibility
Source: Front Immunol. 2023 Feb 23;14:1082144. doi: 10.3389/fimmu.2023.1082144 (PMC9997099; doi:10.3389/fimmu.2023.1082144)
Supplement: Supplementary file 1 [file Table_1.docx]

Supplementary **Table 1. The details of genes used for bioinformatics analysis**

| **Ensembl gene ID** | **Official Gene ID** | **Complete name of gene** | **Species** |
| --- | --- | --- | --- |
| [ENSBTAG00000003255](http://www.genecards.org/cgi-bin/carddisp.pl?gene=ENSBTAG00000003255) | CD4 | CD4 molecule(CD4) | Bos taurus |
| [ENSBTAG00000012280](http://www.genecards.org/cgi-bin/carddisp.pl?gene=ENSBTAG00000012280) | PGLYRP2 | peptidoglycan recognition protein 2(PGLYRP2) | Bos taurus |
| [ENSBTAG00000019716](http://www.genecards.org/cgi-bin/carddisp.pl?gene=ENSBTAG00000019716) | CXCL8 | C-X-C motif chemokine ligand 8(CXCL8) | Bos taurus |
| [ENSBTAG00000051891](http://www.genecards.org/cgi-bin/carddisp.pl?gene=ENSBTAG00000051891) | CXCL8 | C-X-C motif chemokine ligand 8(CXCL8) | Bos taurus |
| [ENSBTAG00000003305](http://www.genecards.org/cgi-bin/carddisp.pl?gene=ENSBTAG00000003305) | NCF1 | neutrophil cytosolic factor 1(NCF1) | Bos taurus |
| [ENSBTAG00000007531](http://www.genecards.org/cgi-bin/carddisp.pl?gene=ENSBTAG00000007531) | NCF4 | neutrophil cytosolic factor 4(NCF4) | Bos taurus |
| [ENSBTAG00000018103](http://www.genecards.org/cgi-bin/carddisp.pl?gene=ENSBTAG00000018103) | HMGB1 | high mobility group box 1(HMGB1) | Bos taurus |
| [ENSBTAG00000001246](http://www.genecards.org/cgi-bin/carddisp.pl?gene=ENSBTAG00000001246) | ATP1A1 | ATPase Na+/K+ transporting subunit alpha 1(ATP1A1) | Bos taurus |
| [ENSBTAG00000005574](http://www.genecards.org/cgi-bin/carddisp.pl?gene=ENSBTAG00000005574) | CLU | clusterin(CLU) | Bos taurus |
| [ENSBTAG00000037533](http://www.genecards.org/cgi-bin/carddisp.pl?gene=ENSBTAG00000037533) | C4A | complement component 4A(C4A) | Bos taurus |
| [ENSBTAG00000012210](http://www.genecards.org/cgi-bin/carddisp.pl?gene=ENSBTAG00000012210) | C5 | complement C5(C5) | Bos taurus |
| [ENSBTAG00000026753](http://www.genecards.org/cgi-bin/carddisp.pl?gene=ENSBTAG00000026753) | CXCR1 | chemokine (C-X-C motif) receptor 1(CXCR1) | Bos taurus |
| [ENSBTAG00000038042](http://www.genecards.org/cgi-bin/carddisp.pl?gene=ENSBTAG00000038042) | CXCR2 | C-X-C motif chemokine receptor 2(CXCR2) | Bos taurus |
| [ENSBTAG00000015032](http://www.genecards.org/cgi-bin/carddisp.pl?gene=ENSBTAG00000015032) | CD14 | CD14 molecule(CD14) | Bos taurus |
| [ENSBTAG00000016864](http://www.genecards.org/cgi-bin/carddisp.pl?gene=ENSBTAG00000016864) | LBP | lipopolysaccharide binding protein(LBP) | Bos taurus |
| [ENSBTAG00000018137](http://www.genecards.org/cgi-bin/carddisp.pl?gene=ENSBTAG00000018137) | A2M | alpha-2-macroglobulin(A2M) | Bos taurus |
| [ENSBTAG00000002635](http://www.genecards.org/cgi-bin/carddisp.pl?gene=ENSBTAG00000002635) | PGLYRP1 | peptidoglycan recognition protein 1(PGLYRP1) | Bos taurus |
| [ENSBTAG00000012047](http://www.genecards.org/cgi-bin/carddisp.pl?gene=ENSBTAG00000012047) | JAK2 | Janus kinase 2(JAK2) | Bos taurus |
| [ENSBTAG00000054761](http://www.genecards.org/cgi-bin/carddisp.pl?gene=ENSBTAG00000054761) | MBL1 | mannose binding lectin 1(MBL1) | Bos taurus |
| [ENSBTAG00000007049](http://www.genecards.org/cgi-bin/carddisp.pl?gene=ENSBTAG00000007049) | MBL2 | mannose binding lectin 2(MBL2) | Bos taurus |
| [ENSBTAG00000009496](http://www.genecards.org/cgi-bin/carddisp.pl?gene=ENSBTAG00000009496) | STAT5A | signal transducer and activator of transcription 5A(STAT5A) | Bos taurus |
| [ENSBTAG00000010125](http://www.genecards.org/cgi-bin/carddisp.pl?gene=ENSBTAG00000010125) | STAT5B | signal transducer and activator of transcription 5B(STAT5B) | Bos taurus |
| [ENSBTAG00000005503](http://www.genecards.org/cgi-bin/carddisp.pl?gene=ENSBTAG00000005503) | PRMT2 | protein arginine methyltransferase 2(PRMT2) | Bos taurus |
| [ENSBTAG00000020569](http://www.genecards.org/cgi-bin/carddisp.pl?gene=ENSBTAG00000020569) | CACNA2D1 | calcium voltage-gated channel auxiliary subunit alpha2delta 1(CACNA2D1) | Bos taurus |
| [ENSBTAG00000013955](http://www.genecards.org/cgi-bin/carddisp.pl?gene=ENSBTAG00000013955) | TRAPPC9 | trafficking protein particle complex subunit 9(TRAPPC9) | Bos taurus |
| [ENSBTAG00000007273](http://www.genecards.org/cgi-bin/carddisp.pl?gene=ENSBTAG00000007273) | TF | transferrin(TF) | Bos taurus |
| [ENSBTAG00000012808](http://www.genecards.org/cgi-bin/carddisp.pl?gene=ENSBTAG00000012808) | MASP2 | MBL associated serine protease 2(MASP2) | Bos taurus |
| [ENSBTAG00000005989](http://www.genecards.org/cgi-bin/carddisp.pl?gene=ENSBTAG00000005989) | LAP3 | leucine aminopeptidase 3(LAP3) | Bos taurus |
| [ENSBTAG00000005397](http://www.genecards.org/cgi-bin/carddisp.pl?gene=ENSBTAG00000005397) | CD46 | CD46 molecule(CD46) | Bos taurus |
| [ENSBTAG00000006240](http://www.genecards.org/cgi-bin/carddisp.pl?gene=ENSBTAG00000006240) | TLR4 | toll like receptor 4(TLR4) | Bos taurus |
| [ENSBTAG00000008008](http://www.genecards.org/cgi-bin/carddisp.pl?gene=ENSBTAG00000008008) | TLR2 | toll like receptor 2(TLR2) | Bos taurus |
| [ENSBTAG00000017704](http://www.genecards.org/cgi-bin/carddisp.pl?gene=ENSBTAG00000017704) | ABCG2 | ATP binding cassette subfamily G member 2(ABCG2) | Bos taurus |
