# Supplementary material for: Genetic polymorphisms in immune- and inflammation-associated genes and their association with bovine mastitis resistance/susceptibility
Source: Front Immunol. 2023 Feb 23;14:1082144. doi: 10.3389/fimmu.2023.1082144 (PMC9997099; doi:10.3389/fimmu.2023.1082144)
Supplement: Supplementary file 2 [file Table_2.docx]

Supplementary Table 2. Biological signaling pathways

| **Biological Signaling pathways** | **P-value** | **Genes** |
| --- | --- | --- |
| bta05417:Lipid and atherosclerosis | 3.55E-06 | CXCL8, NCF1, NCF4, LBP, CD14, JAK2, TLR4, TLR2 |
| bta05171:Coronavirus disease - COVID-19 | 1.06E-05 | C4A, C5, CXCL8, MASP2, TLR4, MBL1, MBL2, TLR2 |
| bta04936:Alcoholic liver disease | 6.63E-05 | C4A, C5, CXCL8, LBP, CD14, TLR4 |
| bta05133:Pertussis | 6.64E-05 | C4A, C5, CXCL8, CD14, TLR4 |
| bta05140:Leishmaniasis | 6.64E-05 | NCF1, NCF4, JAK2, TLR4, TLR2 |
| bta05161:Hepatitis B | 1.28E-04 | STAT5A, STAT5B, CXCL8, JAK2, TLR4, TLR2 |
| bta05134:Legionellosis | 6.17E-04 | CXCL8, CD14, TLR4, TLR2 |
| bta05162:Measles | 0.001043551 | STAT5A, STAT5B, CD46, TLR4, TLR2 |
| bta04217:Necroptosis | 0.001614371 | STAT5A, STAT5B, HMGB1, JAK2, TLR4 |
| bta05235:PD-L1 expression and PD-1 checkpoint pathway in cancer | 0.002381852 | CD4, JAK2, TLR4, TLR2 |
| bta05152:Tuberculosis | 0.002597295 | LBP, CD14, JAK2, TLR4, TLR2 |
| bta04933:AGE-RAGE signaling pathway in diabetic complications | 0.003089129 | STAT5A, STAT5B, CXCL8, JAK2 |
| bta05146:Amoebiasis | 0.004424397 | CXCL8, CD14, TLR4, TLR2 |
| bta05144:Malaria | 0.013123304 | CXCL8, TLR4, TLR2 |
| bta05221:Acute myeloid leukemia | 0.016136375 | STAT5A, STAT5B, CD14 |
| bta04061:Viral protein interaction with cytokine and cytokine receptor | 0.030166865 | CXCL8, CXCR1, CXCR2 |
| bta05323:Rheumatoid arthritis | 0.036883215 | CXCL8, TLR4, TLR2 |
| bta05132:Salmonella infection | 0.037930511 | CXCL8, CD14, TLR4, TLR2 |
| bta05145:Toxoplasmosis | 0.042097545 | JAK2, TLR4, TLR2 |
| bta04935:Growth hormone synthesis, secretion and action | 0.045490295 | STAT5A, STAT5B, JAK2 |
| bta05142:Chagas disease | 0.047573622 | CXCL8, TLR4, TLR2 |
| bta04072:Phospholipase D signaling pathway | 0.071019307 | CXCL8, CXCR1, CXCR2 |
| bta05135:Yersinia infection | 0.074277553 | CD4, CXCL8, TLR4 |
